# Epidemiological Profiles of Human Rabies Cases in Tunisia Between 2000 and 2022

**DOI:** 10.3390/v17070966

**Published:** 2025-07-10

**Authors:** Amal Ayachi, Rym Benabdallah, Aida Bouratbine, Karim Aoun, Jihen Bensalem, Nourhen Basdouri, Samia Benmaiz, Farah Bassalah, Chaima Nouioui, Mohamed Soltani, Khaled Ghouili, Zied Bouslema, Habib Kharmechi, Mariem Handous

**Affiliations:** 1 Faculty of Medicine of Tunis, University of Tunis El Manar, Tunis 1007, Tunisia; a.amalayachi@gmail.com; 2 Laboratory of Medical Parasitology, Biotechnology and Biomolecules, Institut Pasteur de Tunis, University of Tunis El Manar, Tunis 1002, Tunisia; rym.benabdallah@fmt.utm.tn (R.B.); aida.bouratbine@pasteur.rns.tn (A.B.); karim.aoun@pasteur.tn (K.A.); 3 Laboratory of Epidemiology and Veterinary Microbiology, Group of Virology, Institut Pasteur de Tunis, University of Tunis El Manar, Tunis 1002, Tunisia; jihen.bensalem@pasteur.tn (J.B.); samia.benmaiez@pasteur.tn (S.B.); farahbassalah3011@gmail.com (F.B.); chaimanouioui@gmail.com (C.N.); medsoltani836@gmail.com (M.S.); khaledghouili2017@gmail.com (K.G.); ziedbouslama80@gmail.com (Z.B.); 4 Rabies Laboratory, Institut Pasteur de Tunis, Tunis 1002, Tunisia; 5 Independent Researcher, Tunis 1000, Tunisia; basdourinourhen@gmail.com (N.B.); h.kharmachi@yahoo.fr (H.K.)

**Keywords:** rabies, zoonosis, clinical virology

## Abstract

In Tunisia, rabies is endemic and represents a significant public health issue. The objectives of our study were to describe the epidemiological and clinical profiles of human rabies cases and report the risk factors associated with their occurrence. We conducted a retrospective, descriptive, and analytical study of human rabies cases confirmed at the Rabies Laboratory of the Pasteur Institute in Tunis from January 2000 to November 2022. Temporal–spatial, sociodemographic, and clinical variables and factors related to the exposure context, post-exposure, and response were collected for each patient. A total of 58 human rabies cases were identified. The governorates of Kairouan and Nabeul were the most affected, with a predominance of rural areas (77%, 34/44). The highest number of cases was recorded between May and November (74%, 43/58). The cases predominantly involved males, with the most affected age group being individuals aged from 31 to 59 years (30%, 17/57). Rabies transmission was primarily due to dogs (86%, 43/50) and a single bite (55%, 32/58). After an average incubation period of 60.3 days, hydrophobia and behavioral disturbances were the most common symptoms. This study demonstrates that the risk of human rabies remains present in Tunisia, highlighting the need to improve awareness and post-exposure prophylaxis practices.

## 1. Introduction

Rabies is a fatal zoonotic disease with a neurotropic effect caused by a negative-sense RNA virus of the *Lyssavirus* genus [1]. The disease kills approximately 59,000 people annually worldwide and causes USD 8.6 billion in economic losses, primarily in Africa and Asia [2,3]. In 99% of human rabies cases, rabies is transmitted by dogs [3].

In response to this alarming situation, the World Health Organization (WHO), the World Organization for Animal Health (WOAH), the Food and Agriculture Organization (FAO), and the Global Alliance for Rabies Control (GARC) formed a strategic plan, “Zero by 30”, to assist endemic countries in eliminating human deaths from dog-mediated rabies by 2030. This program aims to encourage the preparation of sustainable national “One Health” strategies to eliminate rabies, integrating human and animal health approaches in an intersectoral and coordinated manner [4].

Similar to other low- and middle-income countries, rabies continues to be enzootic and endemic in Tunisia, representing a significant public health problem. The recent history of rabies in Tunisia can be divided into five phases, each marked by variations in the incidence of both animal and human disease [5]. The first period, from 1960 to 1981, was characterized by the absence of national mass dog vaccination campaigns. The second period, from 1982 to 1987, saw the implementation of a national anti-rabies program. The primary objectives of this program were to ensure a high annual vaccination coverage rate in the dog population, reduce the incidence of animal rabies, raise public awareness, manage waste effectively, and prevent human rabies cases, with the ultimate goal of eliminating the disease. The third period, from 1987 to 1992, was marked by a resurgence of both canine and human rabies following the cessation of mass dog vaccination. The fourth period, from 1992 to 2011, was characterized by the revival of vaccination campaigns, leading to a reduction in disease activity. Since 2011, rabies has entered its fifth phase, characterized by an alarming resurgence of both animal and human rabies [6]. Our study is situated between the latter two periods and, thus, aims to provide an epidemiological profile of human rabies from January 2000 to November 2022, studying potential differences between these periods.

Rabies remains a neglected tropical disease, and efforts should require a public health priority given the concerning regional and national situation. However, studies on human rabies—whether descriptive or analytical—remain very limited in Tunisia, which motivated our interest in this work. The objectives of our study were to describe the epidemiological and clinical profiles of human rabies cases between 2000 and 2022 and to report the factors associated with the occurrence of human rabies cases.

## 2. Materials and Methods

This is a retrospective, descriptive, and analytical study that relies on data collection from human rabies cases diagnosed at the Rabies Laboratory of the Pasteur Institute in Tunis (IPT) between January 2000 and November 2022. The Rabies Laboratory at IPT is the sole national reference laboratory responsible for diagnosing both human and animal rabies, following WHO [7] and WOAH guidelines [8].

For ante-mortem diagnosis, the required samples include serum, cerebrospinal fluid, corneal impression, and/or saliva (three samples collected at 3 h intervals). The laboratory employs various reference techniques, including Fluorescent Antibody Test (FAT), Rabies Tissue Culture Infection Test (RTCIT), Rapid Fluorescent Focus Inhibition Test (RFFIT), Fluorescent Antibody Virus Neutralization test (FAVN), and Reverse-Transcription Polymerase Chain Reaction (RT-PCR).

For post-mortem diagnosis, tissue samples from the hippocampus, medulla oblongata, cerebellum, and cerebral cortex are used. The primary techniques in post-mortem diagnosis include direct immunofluorescence and viral isolation in cell culture.

### 2.1. Data Collection

All samples sent to the Rabies Laboratory are accompanied by a form containing details about the exposed patient and the exposure context, provided by the requesting physician. Any missing information (age and origin of the patient, information about the contaminating animal, PEP protocol, etc.) is completed by the laboratory team upon receipt of the sample, in coordination with the requesting physician, to ensure accurate patient record keeping.

Using a pre-established study form (Table S1: Data Collection Form for Human Rabies Cases in Tunisia Between 2000 and 2022), data from the included patient records were collected, covering the following characteristics:

Epidemiological characteristics: Year, season, month, governorate, age, and gender.

Exposure context characteristics: Date of exposure, contact with a suspected animal, type of contact, location, and number of lesions.

Animal characteristics: Species, ownership status, vaccination status, and laboratory diagnosis date.

Post-exposure characteristics: Medical care sought and post-exposure prophylaxis (PEP) course.

Clinical characteristics: Incubation period, symptom nature, and disease progression.

Biological diagnostic characteristics: Time between clinical diagnosis and sampling and type of sample (ante- or post-mortem).

In Tunisia, PEP is based on the following four protocols derived from the WHO guidelines and based on Integrated Bite Case Management (IBCM):

− Protocol A1: Day 0 (D0): Rabies Immunoglobulin (RIG) by wound infiltration +1 dose Rabies Vaccine (RV) via IntraMuscular Route, D3: 1 RV, D7: depends on animal observation
− Protocol A2: D0: 2 doses RV, D7: depends on animal observation
− Protocol B1: D0: RIG + 1 RV, D3: 1 RV, D7: 1 RV, D14: 1 RV, D28: 1 RV
− Protocol B2: D0: 2 RV, D7: 1 RV, D21: 1 RV

### 2.2. Data Analysis

Based on data from 2011, when a rise in animal rabies cases was observed, we divided the study population into the following two groups: the first group consisted of human rabies cases from 2000 to 2011, and the second group included cases from 2012 to 2022. To compare data before and after this date, the variable was divided into two categories.

To calculate the incidence of human rabies cases, demographic data from the National Institute of Statistics were used.

### 2.3. Statistical Analysis

Comparisons of means between independent groups were performed using the Student’s *t*-test. Comparisons of percentages between independent groups were carried out using Pearson’s chi-square test and Fisher’s exact test. Comparisons of quantitative variables with multiple categorical variables were conducted using ANOVA. A significance level of 0.05 was used for all statistical tests. Data were entered and analyzed using IBM SPSS Statistics version 26.0.

## 3. Results

### 3.1. Epidemiological Profile of Human Rabies in Tunisia (2000–2022)

#### 3.1.1. Temporal Distribution of Human Rabies

Incidence and Annual Distribution

**Figure 1:**
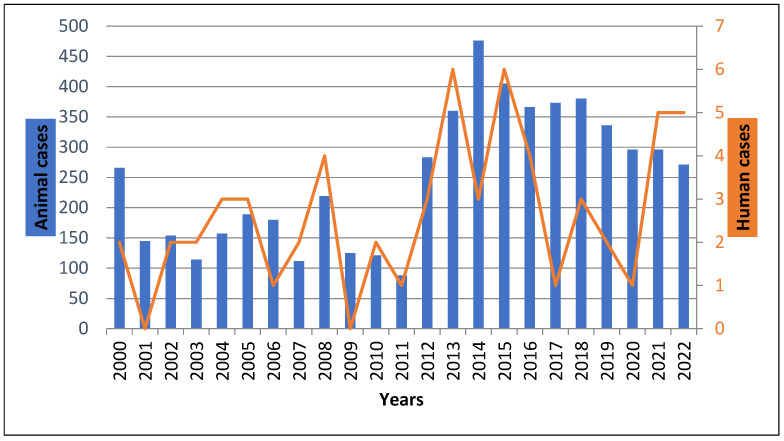
Annual distribution of human and animal rabies cases in Tunisia (2000–2022). The diagrams in blue show animal rabies incidence evolution, whereas the line in orange shows human rabies incidence evolution through the 22 years.

Between 2000 and 2022, 58 human rabies cases were reported, with an annual incidence of 0.02 cases per 100,000 inhabitants. The number of cases per year ranged from 0 to 6, with an average of 2.52 cases per year. The highest recorded incidence occurred in 2013 and 2015, with six cases each. No laboratory-confirmed human rabies cases were diagnosed in 2001, 2009, or 2020 (Figure 1). The annual average of reported cases increased significantly from 1.83 cases per year between 2000 and 2011 to 3.27 cases per year between 2012 and 2022 (*p* < 10^−3^).

Seasonality

During the autumn, 19 (33%, 19/58) cases were recorded. In total, 16 (28%, 16/58) cases occurred in summer, and 13 (22%, 13/58) occurred in spring. In winter, 10 (17%, 10/58) cases were reported. The variation in the number of human rabies cases based on seasonality was not statistically significant (*p* = 0.42).

Monthly Distribution

Eight (14%, 8/58) human rabies cases were reported in May and November. One (2%, 1/58) case was reported in April. The number of human rabies cases varied significantly by month (*p* = 0.034).

#### 3.1.2. Geographic Distribution of Human Rabies

By Governorates

**Figure 2:**
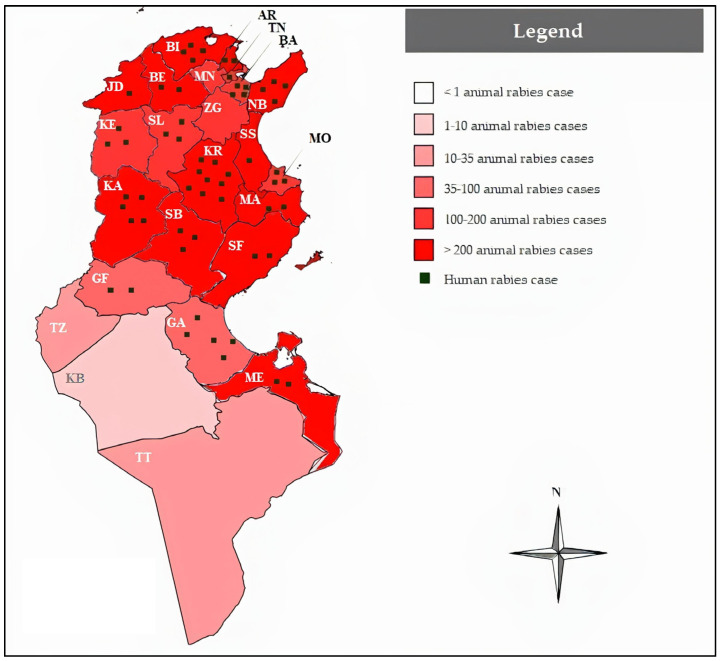
Geographic distribution of human and animal rabies cases in Tunisia (2000–2022). A range of colors and points, respectively, show the distribution of animal and human rabies cases in each governorate during the course of the study period. Ariana (AR), Tunis (TN), Ben arous (BA), Manouba (MN), Zaghouan (ZG), Nabeul (NB), Sousse (SS), Monastir (MO), Mahdia (MA), Sfax (SF), Gabes (GA), Medenine (ME), Tataouine (TT), Kebili (KB), Tozeur (TZ), Gafsa (GF), Sidi Bouzid (SB), Kasserine (KA), Kairouan (KR), Siliana (SL), Kef (KE), Jendouba (JD), Beja (BJ), and Bizerte (BZ).

Throughout the study period, the governorate of Kairouan recorded nine (16%, 9/58) human rabies cases, followed by Nabeul with six (10%, 6/58) cases, and Kasserine with five (9%, 5/58) cases. In total, 6 of the 24 Tunisian governorates—Kebili, Manouba, Sousse, Tataouine, Tozeur, and Zaghouan—reported no laboratory-confirmed human rabies cases from 2000 to 2022. The northeastern and western regions (Tunis, Ariana, Manouba, Ben Arous, Nabeul, Zaghouan, Bizerte, Beja, Siliana, Kef, and Jendouba) accounted for 25 human rabies cases (44%, 25/57), while the central–eastern and western regions (Mahdia, Sfax, Sousse, Monastir, Kasserine, Sidi Bouzid, and Kairouan) recorded 23 cases (40%, 23/57). The southern regions (Kebili, Gabes, Tataouine, Medenine, Tozeur, and Gafsa) reported nine cases (16%, 9/57). Figure 2 illustrates the distribution of human rabies cases during the study period and its relationship with animal rabies cases.

Comparing the 2000–2011 and 2012–2022 periods, governorates that reported zero cases in the first period began to report human rabies cases in the subsequent years. For instance, Jendouba reported one case, Ben Arous reported four, and Mahdia, Ariana, and Sfax reported two cases each. This difference was not statistically significant.

By Type of Environment

Seventy-seven percent (77%, 34/44) of human rabies cases occurred in rural areas. From 2013 onward, the number of urban cases rose from one (period between 2000 and 2012) to nine. Data for the years 2001, 2007, 2009, 2011, and 2020 were missing. The difference in the distribution of cases between rural and urban areas was not statistically significant when comparing the 2000–2011 and 2021–2022 periods (*p* = 0.13).

#### 3.1.3. Sociodemographic Distribution of Human Rabies

Gender

During the study period, 44 men succumbed to human rabies, resulting in a male-to-female ratio of 3.14. The highest number of male cases was observed in 2015 (five cases), while the highest number of female cases occurred in 2022 (three cases). The variation in annual cases by gender was not statistically significant (*p* = 0.66). When comparing the two periods (2000–2011 and 2012–2022), the male predominance remained consistent at 73% (n = 16/22) and 78% (n = 28/36), respectively, with no statistically significant difference (*p* = 0.66).

Age

**Figure 3:**
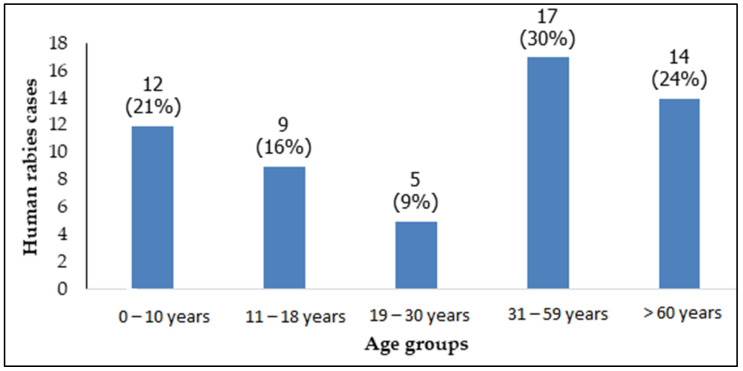
Distribution of human rabies cases by age group.

The average age of the affected patients was 35.12 ± 24.2 years, ranging from 1 to 82 years. From 2000 to 2011, the average age of affected individuals was 30.90 ± 23.9 years, and from 2012 to 2022, it was 37.58 ± 24.4 years, with no statistically significant difference. Seventeen (30%, 17/57) patients affected by rabies were aged between 31 and 59 years. The age distribution of patients is detailed in Figure 3.

Gender and Age

For both genders, the 31–59 years age group was the most affected, with 21% (n = 12/58) of male cases and 9% (n = 5/58) of female cases belonging to this group. Among women aged over 30, 16% (n = 9/58) were affected, compared to 9% (n = 5/58) among women under 30. This difference was not statistically significant (*p* = 0.25).

#### 3.1.4. Distribution of Human Rabies by Characteristics of the Exposing Animal

Animal Species

In 49 (84%, 49/58) cases, contact with an exposing animal was reported. The remaining cases had either no contact or this information was missing. Dogs were responsible for 42 (86%, 42/49) cases of human rabies, followed by cats with 4 (8%, 4/49) cases, 1 cow (2%, 1/49), and 1 sheep (2%, 1/49). Data concerning animal species were missing for one case.

Presence or Absence of an Owner

A total of 18 animals (37%, 18/48) had no owner, 11 (23%, 11/48) had an owner, and for 19 animals (40%, 19/48), the ownership status was unknown. No statistically significant difference was found between the 2000–2010 and 2011–2022 periods (*p* = 0.66).

Outcome of the Exposing Animal

Information on the fate of the exposing animal was available for 13 (27%, 13/49) of the animals involved. Seven were diagnosed with rabies following laboratory testing, and six were euthanized by their owners without a laboratory confirmation of rabies. The fate of the remaining animals was unknown. It is noteworthy that no exposing animal was vaccinated. From 2012 onward, 11 (85%, 11/13) of the exposing animals diagnosed with rabies (clinically and/or in the laboratory) were responsible for human rabies cases. The difference between the 2000–2011 and 2012–2022 periods was statistically significant (*p* = 0.04).

### 3.2. Clinical Profile of Human Rabies During the Period 2000–2022

#### 3.2.1. Distribution of Human Rabies According to Clinical Characteristics

Nature and Site of Contact with the Exposing Animal

Thirty-two patients (55%, 32/58) were infected through a single bite. The head and neck were the contact sites in 19% (11/58) of cases, while the site of exposure was unknown for 60% (35/58) of patients (Table 1).

Nature, Site of Exposure, and Age of Affected Patients

Bites were the primary cause of human rabies across all patient age groups. No significant association was found between the type of contact with the exposing animal and patient age category (*p* = 0.44).

**Figure 4:**
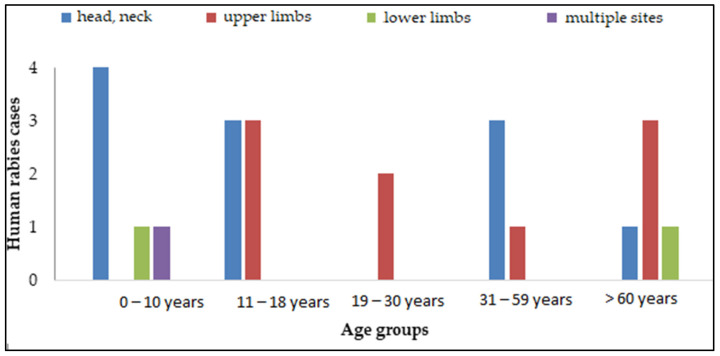
Distribution of exposure site by age group.

Patients aged from 11 to 59 years were not bitten on the lower limbs. No patients aged from 19 to 30 years were bitten on the head and neck, whereas head and neck injuries were predominant in children under 10 years old. No statistically significant relationship was found between age group and site of exposure (*p* = 0.36) (Figure 4).

Incubation Period of Human Rabies

The mean incubation period was 60.3 ± 69.5 days, ranging from 13 days to 1 year, with a median of 30.5 days.

From 2000 to 2011, the mean incubation period was 91 days, whereas it was 35.08 days from 2012 to 2022, with no statistically significant difference (*p* = 0.06).

Incubation Period and Site of Contact with the Exposing Animal

The shortest incubation period (13 days) was observed in cases of head and neck injuries, while the longest (1 year) was observed for lower limb injuries. The mean incubation period was significantly associated with the site of exposure (*p* = 0.04).

Clinical Manifestations of Human Rabies

Patients exhibited a wide range of clinical signs. Table 2 presents the observed clinical manifestations in descending order of occurrence.

#### 3.2.2. Post-Exposure Measures

Wound Washing and Disinfection

Among all patients, seven (12%, 7/58) washed and disinfected the wound site after the bite contact, while two (3%, 2/58) did not. For the remaining patients, this information was unavailable.

Healthcare Facility Consultation

In total, 23 (40%, 23/58) patients did not seek medical care, while 19 (33%, 19/58) consulted a healthcare facility after exposure. For the remaining 27% (16/58), no information was recorded. The proportion of patients seeking post-exposure care was significantly higher in rural areas (37%, 21/58) than in urban areas (11%, 6/58) (*p* = 0.027).

Utilization of Post-Exposure Prophylaxis (PEP)

**Figure 5:**
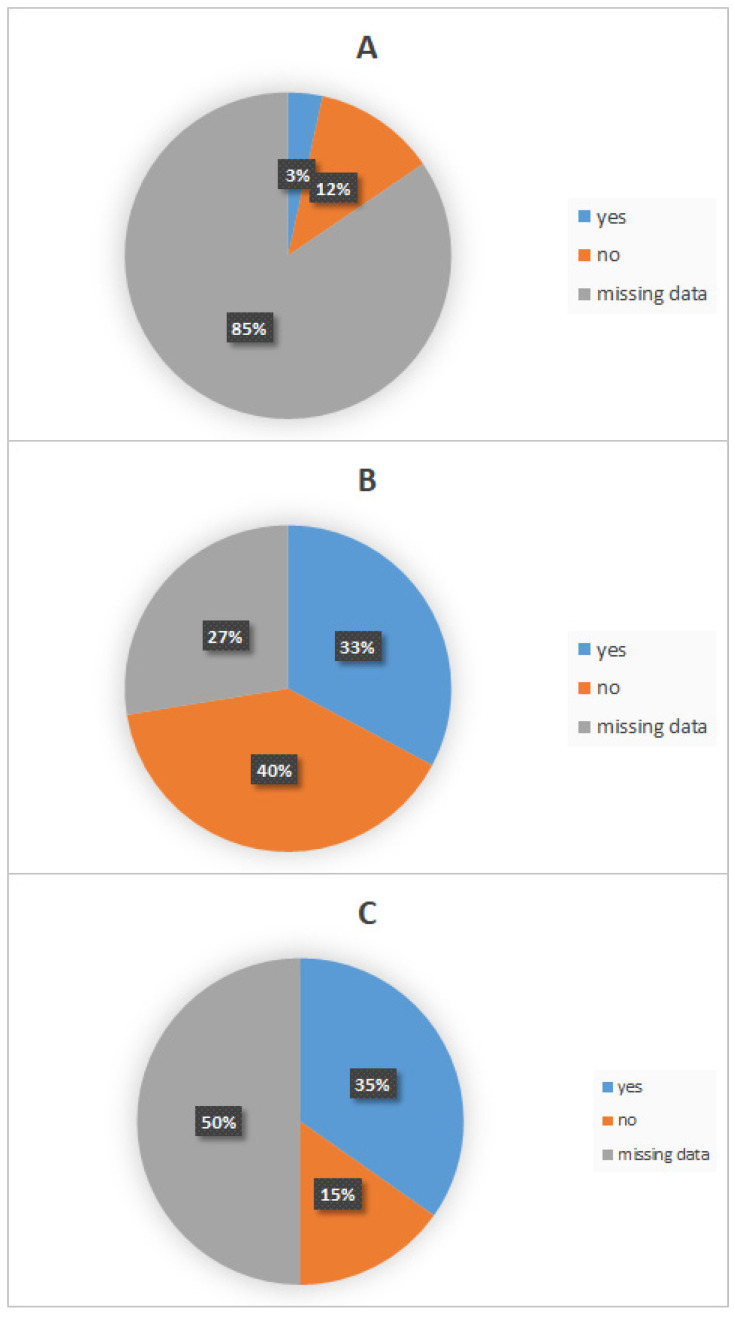
Use of different preventive measures following rabies exposure. Pie charts represent proportions of recourse to (**A**) wound washing and disinfection, (**B**) healthcare facility consultation, and (**C**) utilization of post-exposure prophylaxis.

Sixteen (28%, 16/58) patients received PEP, while seven (12%, 7/58) did not. For the remaining 40% (23/58), data were unavailable. No statistically significant difference was observed between the two study periods (2000–2011 vs. 2012–2022) (*p* = 0.07) (Figure 5).

Time Interval Between Exposure and Post-Exposure Prophylaxis Administration

Among patients who received PEP, 64% (9/14) received it within 24 h of exposure, 22% (3/14) after 24 h, 7% (1/14) on day 4, and 7% (1/14) beyond day 4, with some delaying treatment up to 7 days.

Post-Exposure Prophylaxis Protocol

Among patients who received PEP, 92% (12/13) received a combination of RIG and the vaccine, while 8% (1/13) received the vaccine alone. Twelve patients followed protocol B1, while one patient initially started protocol A1 but was switched to B1 after the exposing animal died. The PEP regimen was not completed in eight (62%, 8/13) patients due to missed vaccine doses, as follows: failure to receive RV on day 3 or day 7, discontinuation of protocol B1 after the day 14 dose, or symptom onset before the day 28 dose. For five (38%, 5/13) patients, the full regimen was completed. The head and neck were the sites of exposure. The study also noted the improper administration of RIG in some cases, including the absence of RIG administration, intramuscular injection instead of local infiltration, or an insufficient dosage. Additionally, some patients underwent wound suturing.

#### 3.2.3. Disease Progression and Outcome

Time from Symptom Onset to Death

Among the 58 rabies patients, 57 (98%, 57/58) died. The mean disease duration was 4.96 ± 4.5 days (range: 0–16 days). Thirty-six (74%, 36/49) patients died within 7 days of symptom onset, while eight (16%, 8/49) died within hours of symptom onset.

Laboratory Diagnosis

Biological diagnosis was performed post-mortem in 55 (95%, 55/58) cases and ante-mortem in 3 (5%, 3/58) cases. The first ante-mortem diagnosis was carried out with FAT on CSF, the second with RTCIT on saliva, and the third with FAVN on CSF and serum.

Time from Clinical Diagnosis to Sample Collection

The mean interval between clinical diagnosis and sample collection was 5.45 ± 4.47 days (range: 0–20 days). In 4% (2/57) of cases, sampling was performed on the same day as clinical diagnosis.

Time from Sample Collection to Laboratory Confirmation

The mean time between sample collection and laboratory confirmation was 0.89 ± 2.1 days (range: 0–15 days). In 31 (55%, 31/57) cases, confirmation was obtained on the same day as sample collection, and in 98% (56/57) of cases, it was achieved within one week.

## 4. Discussion

### 4.1. Temporal Distribution

Between January 2000 and November 2022, a total of 58 human rabies cases were reported, with an average annual incidence of 2.52 cases and a mortality rate of 98% (57/58). We reported one case of rabies survival in which specific antibodies were detected in the cerebrospinal fluid (CSF) and serum [9].

Although a decrease in human rabies cases was observed compared to the 1983–2000 period—which was marked by a major peak of 25 human rabies deaths and 581 animal rabies cases in 1992—this previous peak was attributed to the suspension of mass dog vaccination campaigns between 1988 and 1992 [5]. However, the year 2011 marked a turning point in the temporal distribution of rabies in Tunisia, leading to a new phase characterized by a resurgence of animal rabies, which peaked at 377 cases in that year, along with a concurrent increase in human rabies cases [5]. In agreement with these observations, our study also showed that the annual number of human rabies cases began rising in 2011, reaching two peaks of six cases in 2013 and 2015, followed by additional peaks of five cases in both 2021 and 2022. Moreover, the average number of human rabies cases significantly increased (*p* < 10^−3^) when comparing the two periods, 2000–2011 and 2012–2022.

The estimated annual incidence was 0.02 per 100,000 inhabitants, which is lower than the WHO’s estimated range of 0.038 to 0.19 per 100,000 inhabitants [3]. This discrepancy suggests a likely underestimation of the actual number of cases. Underreporting of human rabies deaths has been documented in Africa (by a factor of 160) and Asia (by a factor of 20) [10], likely due to differences in surveillance system effectiveness [11]. This issue was reflected in our study by the absence of laboratory-confirmed cases in 2020, despite a clinically recorded rabies case [12].

Although rabies is a notifiable disease, human rabies surveillance in Tunisia operates as a passive surveillance system, similar to most endemic countries, which affects the accuracy of recorded data.

Analysis of the monthly distribution of human rabies cases revealed a statistically significant variation (*p* = 0.034), with 74% (43/58) of cases occurring between May and November. A similar seasonal pattern was observed in a 54-year study conducted in China, the second-most rabies-endemic country in Asia [13]. This period coincides with Tunisia’s warmer months, during which people spend more time outdoors wearing lighter clothing, potentially increasing exposure to animal bites.

Additionally, the observed two-month average incubation period in our study and the peak months of canine rabies cases (April and May) recorded in IPT reports further explain the rise in human rabies cases between May and November. Throughout the study period, no significant seasonal variation in human rabies cases was observed.

### 4.2. Geographical Distribution

The northern governorates accounted for 44% (26/58) of human rabies cases, followed by the central governorates with 40% (23/58), while the southern governorates reported only 16% (9/58) of cases. A parallel can be drawn between the geographical distribution of human and animal rabies [14]. However, it is crucial to consider that infected dogs may travel during the silent incubation period, potentially spreading rabies to previously unaffected areas. This scenario was exemplified in Bali, Indonesia, where the island remained rabies-free until the outbreak of 2008, which led to 141 human deaths by the end of 2012 [15].

Our study suggests that urban areas are not primary hotspots for rabies, with rural areas being predominantly affected (77%, 34/44). However, a notable increase in reported animal attacks in urban settings was observed, as follows: only two cases occurred over a 16-year period (2000–2015), whereas eight cases were recorded between 2016 and 2022. It has been demonstrated that rabies transmission can extend into peri-urban and urban areas due to human-mediated dog transportation [16], as well as changes in the Tunisian population’s interaction with animals. The molecular characterization of rabies virus isolates from brain tissue samples of infected dogs revealed geographical variability, identifying the following two distinct lineages: one localized in the northwest regions (NW lineage) and another spanning the northeast, central, and northern regions (NCS lineage) [17].

The geographic expansion of human rabies highlights the vulnerability of anti-rabies control efforts in certain governorates, potentially due to weaker engagement in regions that have remained rabies-free for extended periods. Given the heterogeneous distribution of human rabies cases, their temporal instability, and the predominance of cases in rural settings, the key challenge is to enhance the surveillance system for human rabies, animal rabies, and PEP at the local and regional levels, rather than relying predominantly on a broad national-scale approach.

A similar initiative was implemented in Cameroon, where localized efforts led to improved surveillance effectiveness for both human and animal rabies, as well as PEP administration. This approach provides a valuable foundation for future rabies control strategies [18].

### 4.3. Characteristics of Exposed Patients

Similar to findings from several other studies [19,20], our study indicates that men were more affected by rabies than women, with a male-to-female sex ratio of 3.14. This disparity is likely influenced by cultural factors, as men in Tunisia are more frequently engaged in outdoor activities, increasing their risk of exposure to potentially rabid animals.

Human rabies cases were recorded across all age groups in our study, with a predominance among individuals over 31 years old (54%, 31/57). Notably, this age distribution remained relatively stable throughout the study period (2000–2022).

According to the World Health Organization (WHO), although all age groups are susceptible to rabies, children under 15 years old are at the highest risk. On average, 40% of post-exposure prophylaxis (PEP) vaccinations are administered to children aged from 5 to 14 years [21]. However, our findings diverge from WHO reports and other studies [19,22,23], as children and adolescents (0–18 years old) accounted for only 37% (21/57) of human rabies cases, whereas adults represented the majority. While children are more likely to engage with and provoke biting animals and, due to their smaller stature, sustain severe facial injuries, our results suggest that adult populations should be a primary target in rabies control efforts. This shift may be attributed to sociodemographic changes observed in other Asian [24] and African [25] countries.

### 4.4. Characteristics of the Exposing Animal

In Tunisia, rabies primarily affects dogs, accounting for approximately 60% of reported cases [26,27]. This pattern is consistent with broader North African trends, where canine rabies represents around 40% of all rabies cases [5], followed by bovine rabies [28].

At the national level, studies have shown that rabies cases in other animal species tend to occur within a 3 km radius of canine rabies hotspots. A strong correlation has been identified between canine rabies incidence and rabies occurrence in other species [27]. Dogs are the main reservoir for rabies in Tunisia, and wildlife animals are only victims [29]. In our study, when the exposing animal was identified, dogs were responsible for the vast majority of attacks (86%, 42/49).

The mass vaccination of dogs remains the most effective strategy for eliminating canine rabies, establishing herd immunity in reservoir species, and interrupting interspecies and human transmission [5,30]. The WHO estimates that vaccinating 70% of the dog population for seven consecutive years could eliminate rabies from a region [31].

In Tunisia, the dog vaccination coverage rate reached 45% in 2000, but after stabilizing, it significantly declined, falling to 9.3% in 2011, coinciding with a rise in canine rabies cases. This drop was largely attributed to political and social instability, which weakened disease control and surveillance efforts [5].

A recent study in northwestern Tunisia found that stray dogs accounted for 14% of the urban dog population and 4% of the rural dog population. These stray dogs were predominantly concentrated around waste disposal sites and slaughterhouses in both urban and rural settings [32]. Poor domestic waste management in some areas has further exacerbated stray dog proliferation and facilitated viral transmission among dogs [5], complicating control measures.

Additionally, the study found that 51.1% of rural dogs and 31.4% of urban dogs were “free-roaming” despite having owners [32]. According to the WOAH, any dog—whether owned or not—that is not under direct human supervision is considered a free-roaming or stray dog [33]. This distinction is crucial, as even owned dogs may temporarily become strays due to abandonment.

Our study found that 23% (11/48) of exposing animals had known owners, while 37% (18/48) were identified as ownerless dogs. However, for this latter group, it was not possible to distinguish between genuinely stray dogs and free-roaming but owned dogs. This distinction is critical, as the inaccessibility of parenteral vaccination to free-roaming dogs contributes to inadequate vaccination coverage, undermining herd immunity within the free-roaming dog population [34].

### 4.5. Clinical Characteristics

More than half of the patients in our study were infected through a single bite (55%, 32/58). A single bite is sufficient to transmit rabies, as saliva is the primary medium for rabies virus secretion and spread.

Bites combined with scratches accounted for 20% (11/57) of fatal cases, followed by scratches alone in 9% (5/58) of cases, and multiple bites in only 5% (3/58) of cases. One patient, a shepherd, was infected through the contact of contaminating saliva with wounds on the arm when handling the animal’s mouth. In 9% (5/58) of cases, the nature of the exposure was unknown, as these patients were either unconscious upon admission or their relatives were unaware of the circumstances of the attack.

It is important to note that rabies transmission can occur through routes other than bites or scratches, which may go unnoticed or remain unidentified. These include aerosol transmission (e.g., in bat-infested caves), handling infected carcasses, and the contamination of open wounds, abrasions, or mucous membranes with infected saliva or neural tissue [35].

Human-to-human transmission is rare but has been reported via organ and tissue transplantation, particularly corneal transplantation. This risk has led some low- and middle-income countries to implement rabies screening for organ donors, given that rabies diagnosis can be overlooked in non-endemic regions [36,37]. In Iran, two patients contracted rabies after receiving corneal transplants from an undiagnosed rabies-infected donor, prompting the country to introduce systematic screening for corneal donors [38].

In Tunisia, donor screening for rabies has not yet been implemented. However, in the context of our study, the recorded rabies cases did not involve any organ transplantation.

Exposure primarily occurred on the face and neck (19%, 11/58) and upper limbs (16%, 9/58), though site information was missing for 60% (35/58) of cases. These areas are more accessible to biting animals and are associated with more severe injuries and shorter incubation periods [39]. This finding aligns with a study conducted in Bousalem, Jendouba Governorate [40]. In 60% (35/58) of cases, the site of exposure was unknown, likely due to altered consciousness or a prolonged incubation period.

Bites on the face and neck had the shortest mean incubation period (29.1 days), compared to bites on the upper limbs (105.2 days) and lower limbs (180 days). Multiple-bite exposures had the shortest incubation period overall (23 days). We found a statistically significant association between incubation period and exposure site (*p* = 0.04), consistent with other studies [41,42].

Due to the retrospective nature of our study, we were unable to assess incubation duration based on the severity of exposure, as categorized by the WHO (Category II or III), despite evidence suggesting a significant correlation [43].

The mean incubation period was 60.3 ± 69.5 days, with a median of 30.5 days. In 43% (25/58) of cases, disease onset occurred between one and three months post-exposure, similar to findings in other studies [23,43,44]. A large cohort study in India reported that 90% of rabies cases developed within six months [45]. A more recent study of 1839 rabies cases found that incubation ranged from three to twelve months in 42.8% of cases [40]. Before migrating from the peripheral nervous system to the central nervous system, the rabies virus persists in muscle tissue during the incubation period, where it undergoes low-level replication or remains in a latent state [34,45].

Only four patients (10%, 4/42) in our study had a prolonged incubation period (>3 months). However, we cannot rule out the possibility of re-exposure, as these patients had forgotten the exact date of their exposure. In rabies-endemic regions, recurrent exposures are common.

Extremely prolonged incubation periods have been documented, including 4 years [43], 6.5 years [46], and even exceptionally long periods of 25 years [47] and 27 years [41]. In imported rabies cases among migrants and travelers from endemic areas, long incubation periods have posed diagnostic challenges in non-endemic countries. Molecular virology studies have traced viral migration patterns in such cases, though the precise mechanisms underlying prolonged latency remain unclear [47,48]. These findings highlight the importance of considering long-term travel histories in patients presenting with unexplained encephalitis.

Variable incubation periods with an extended duration are also observed in animal rabies, contributing to the persistence of rabies enzootics [49].

Paresthesia, pain, and/or itching at the site of exposure are among the earliest neurological signs of rabies, occurring in approximately 50% of paralytic cases and 30% of furious cases [50]. These symptoms are thought to be associated with inflammation of the dorsal root ganglion or cranial sensory ganglia [51]. However, in our study, such early signs were reported in only 14% (7/51) of patients.

These symptoms characterize the prodromal phase of rabies, which can also include other non-specific signs. In our cohort, fever was the most frequently observed non-specific symptom (35%, 18/51), followed by vomiting (22%, 11/51), asthenia (8%, 4/51), headaches (6%, 3/51), arthro-myalgia (6%, 3/51), and dizziness (2%, 1/51). Recognizing this early stage is crucial, as failure to do so increases the risk of missing a rabies diagnosis.

Hydrophobia and aerophobia, often considered hallmark symptoms of rabies, were not consistently present in our study, occurring in 67% (34/51) and 18% (9/51) of cases, respectively. These figures are notably lower than those reported in a large case series from the Philippines, where hydrophobia was observed in all patients and aerophobia in 95.5% [41], as well as in a study from Bangladesh with similar findings [42]. In our study, behavioral disturbances (65%, 33/51) and fever were relatively frequent (35%, 18/51), and such presentations can lead to rabies being misdiagnosed as a neurological or psychiatric disorder, particularly when exposure history is not actively sought.

Rabies is traditionally classified into the furious form, characterized by predominant hyperactive neurological symptoms, and the paralytic form, marked by neurological deficits. However, in our study, this dichotomy was not always clear-cut, as some patients exhibited overlapping features of both forms.

### 4.6. Post-Exposure Prophylaxis (PEP)

In Tunisia, specialized rabies treatment centers are established across various regions, not just centralized in the capital. Additionally, rabies prophylaxis—including both vaccination and immunoglobulin therapy—is provided free of charge as part of the national rabies control program.

Immediate washing of the wound with soap and running water for at least 15 min, followed by antiseptic application, is a critical step in rabies exposure management. In our study, data on this initial wound care step were largely missing (reported in only seven patients (12%, 7/58)), likely reflecting a lack of awareness regarding its importance. This is concerning, as it is well-documented that one-third of rabies infections could be prevented through proper wound cleaning and disinfection alone [52].

Only 33% (19/58) of patients in our study sought medical care following exposure, and just 28% (16/58) received PEP. Furthermore, only 64% (10/16) of those who sought care did so within the first 24 h post-exposure. However, 7% (1/16) delayed PEP for more than seven days. Previous studies have highlighted persistent inefficiencies and delays in PEP accessibility across various endemic regions [53].

Community awareness programs are essential for educating the public on rabies risks and the necessary steps to take after exposure. A multinational study conducted in Asia involving 4359 participants found that 34% were unaware of rabies, and 30% did not know about the existence of rabies treatment centers. The most common source of information on rabies was friends and family, with healthcare professionals cited as the source in only 26% of cases [54].

Unlike many countries where rural populations face greater challenges in accessing PEP and medical care [22], our study revealed a statistically significant urban–rural disparity in healthcare-seeking behavior (*p* = 0.027). Specifically, 37% (21/58) of rural patients sought medical attention compared to only 11% (6/58) of urban patients. This suggests a potential underestimation of rabies risk among urban populations, highlighting the need for targeted awareness campaigns in these areas.

The use of traditional healers and herbal remedies for rabies treatment remains an observed practice in some regions [42,54]. While rare in Tunisia, some human rabies cases have been linked to such practices. In one documented case, a woman who provided “healing” by spitting on patients later succumbed to rabies. However, such cases have only come to light through post-mortem investigations.

Alarmingly, 62% (7/11) of patients in our study did not complete the full PEP vaccination schedule. Reasons included missed doses, abandonment of the protocol by day 14, or symptom onset before completion. It is crucial to emphasize the necessity of completing the PEP regimen, as it remains the only effective measure to prevent rabies. Usually, the center contact police enforcement to request the return of people who have failed to come for their vaccination appointment after obtaining a mail summons. However, an application that connects all stakeholders, including citizens, in real time may help to increase compliance. Individuals who fail to receive PEP properly and promptly are at a significantly higher risk of developing rabies (Odds Ratio [OR] = 17.33, 95% Confidence Interval [CI]: 6.39–60.83) [55].

Despite optimal PEP administration, rare cases of rabies-related deaths have been reported, often linked to procedural errors [56]. One notable case in Tunisia involved a young girl who developed fatal rabies encephalitis following wound suturing, which may have facilitated viral entry—a factor previously implicated in rabies fatalities despite completed PEP [57]. In our study, wound suturing was observed in multiple cases and was likely a contributing factor to rabies progression, underscoring the need for strict adherence to rabies exposure management protocols.

### 4.7. Response and Diagnostic Challenges

Biological confirmation of suspected or probable human rabies cases remains a challenge in many endemic countries [10], yet it is essential for effective disease surveillance. The morbidity and mortality of rabies are often underestimated due to the absence of simple and sensitive diagnostic methods in some regions [58].

In Tunisia, suspected or probable clinical cases undergo biological confirmation in accordance with WHO recommendations [3]. However, vigilance is required, as false-negative biological samples can occur due to intermittent viral shedding, particularly in ante-mortem samples [59].

During the study period, post-mortem biological testing was performed in the vast majority of cases (95%, 54/57), with clinical diagnosis made, on average, 5.45 ± 4.47 days before sample collection. Early diagnosis is crucial for optimizing case management, as it prevents unnecessary diagnostic procedures and treatments while ensuring the timely prophylactic vaccination of close contacts and healthcare personnel [60]. However, the major diagnostic challenge remains ante-mortem confirmation, which is increasingly relevant, particularly in atypical presentations or paralytic forms that mimic other neurological conditions [50].

Our findings highlight the importance of ante-mortem diagnostic capability, especially since our study showed that the average time between sample collection and biological confirmation was short (0.89 ± 2.1 days), with 55% (31/57) of cases confirmed on the same day. However, the current lack of diagnostic tools for detecting the virus in skin biopsy samples remains a limitation for ante-mortem diagnosis.

In our study, death occurred, on average, 4.96 ± 4.5 days after symptom onset, with cases ranging from 0 to 16 days. The disease course is invariably short, though its duration depends on the clinical form and the nature of medical intervention. In paralytic forms, survival can extend up to two weeks [38], while intensive care management can prolong survival beyond a month [35]. This observation raises questions about the potential for therapeutic approaches to extend the median survival of rabies. However, our study did not specifically analyze the impact of intensive care management on disease duration.

Rabies remains nearly universally fatal, with rare documented cases of survival [61,62,63]. Our study reported one case of survival with severe neurological sequelae—a 7-year-old girl who was bitten on the face by a dog. She presented with an atypical clinical picture, including fever, headaches, ataxia, and behavioral disturbances. Post-exposure prophylaxis was initiated immediately after the bite, and the full vaccination schedule was administered. However, the immunoglobulin serum was not properly applied intralesionally, raising questions about the efficacy of the intervention [9].

## 5. Conclusions

Since 2011, both human and canine rabies cases have shown a concerning increase in Tunisia, with a notable expansion into urban and peri-urban areas. The most affected demographic consists of adult males aged between 31 and 59 years. Domestic dogs remain the primary reservoir and vector of rabies transmission, with over half of human rabies cases resulting from a single bite. While classical clinical manifestations such as aerophobia and hydrophobia may aid diagnosis, they are not consistently observed. Crucially, when post-exposure prophylaxis (PEP) is correctly and promptly administered, it is highly effective in preventing the onset of rabies in humans. Furthermore, sustained annual mass dog vaccination campaigns hold strong potential for the complete elimination of rabies in Tunisia.

Our study underscores the critical need for continued research on human rabies and for monitoring the evolution of the disease over time and across different geographic and socio-political contexts. Given the increasing migration from rabies-endemic countries, studying imported rabies cases in Tunisia is becoming particularly relevant. Expanding the scope of research and ensuring regular epidemiological assessments would contribute to more accurate data, ultimately strengthening surveillance, prevention, and post-exposure management strategies.

Lessons from countries that have successfully eradicated rabies highlight the importance of a multidisciplinary One Health approach. Collaborative efforts between medical, veterinary, and public health sectors are essential to eliminating canine rabies—the primary reservoir—and breaking the animal-to-human transmission cycle.

Preventing human rabies requires enhanced public education on the risks of the disease and the appropriate actions following exposure. Additionally, healthcare providers must receive adequate training to minimize post-exposure prophylaxis (PEP) failures and improve patient adherence to the full vaccination regimen. Strengthening these areas will be crucial in reducing rabies-related mortality and working toward eventual disease elimination.

## Figures and Tables

**Table 1:** Number of human rabies cases by nature and site of exposure.

Nature of Exposure	Cases Number (%)
Single bite	32/58 (55%)
Bites and scratches	12/58 (20%)
Scratches	5/58 (9%)
Multiple bites	3/58 (5%)
Handling of a sick animal	1/58 (2%)
Unknown (not apparent or not reported)	5/58 (9%)
**Site of Exposure**	**Cases Number (%)**
Head, neck	11/58 (19%)
Upper limbs	9/58 (16%)
Lower limbs	2/58 (3%)
Multiple sites	1/58 (2%)
Unknown (not apparent or not reported)	35/58 (60%)

**Table 2:** Clinical signs observed in decreasing order of occurrence.

Clinical Sign	Cases Number (%)
Hydrophobia	34/51 (67%)
Behavioral disturbances	33/51 (65%)
Fever	18/51 (35%)
Altered consciousness, coma	11/51 (22%)
Vomiting	11/51 (22%)
Aerophobia	9/51(18%)
Psychiatric symptoms (hallucinations, anxiety disorders, insomnia)	9/51 (18%)
Hypersalivation	7/51 (14%)
Paresthesia, paralysis, pain at exposure site	7/51 (14%)
Dysphagia	6/51 (12%)
Dyspnea	5/51 (10%)
Asthenia	4/51 (8%)
Chest–abdominal pain	4/51 (8%)
Headache	3/51 (6%)
Arthralgia, myalgia	3/51 (6%)
Photophobia	3/51 (6%)
Tremors	2/51 (3%)
Cutaneous hyperesthesia	1/51 (2%)
Convulsions	1/51 (2%)
Conjunctival hyperemia	1/51 (2%)
Hematemesis	1/51 (2%)
Vertigo	1/51 (2%)

## Data Availability

The data presented in this study are available on request from the corresponding author due to ethical reasons.

## Supplementary Materials

The following supporting information can be downloaded at: https://www.mdpi.com/article/10.3390/v17070966/s1, Table S1: Data Collection Form for Human Rabies Cases in Tunisia Between 2000 and 2022.

## Abbreviations

The following abbreviations are used in this manuscript:

CSF	CerebroSpinal Fluid
FAO	Food and Agriculture Organization
FAT	Fluorescent Antibody Test
FAVN	Fluorescent Antibody Virus Neutralization test
GARC	Global Alliance for Rabies Control
IBCM	Integrated Bite Case Management
IPT	Pasteur Institute in Tunis
PEP	Post-Exposure Prophylaxis
RFFIT	Rapid Fluorescent Focus Inhibition Test
RIG	Rabies ImmunoGlobulin
RTCIT	Rabies Tissue Culture Infection Test
RT-PCR	Reverse-Transcription Polymerase Chain Reaction
RV	Rabies Vaccine
WHO	World Health Organization
WOAH	World Organization of Animal Health
