# Supplementary material for: Epidemiological Profiles of Human Rabies Cases in Tunisia Between 2000 and 2022
Source: Viruses. 2025 Jul 10;17(7):966. doi: 10.3390/v17070966 (PMC12299497; doi:10.3390/v17070966)
Supplement: Supplementary file 1 [file viruses-17-00966-s001.zip › Table S1.pdf]

Table S1 : Data Collection Form for Human Rabies Cases in Tunisia Between 2000 and 2022

**Field**	**Details**
**Form No.**	…..
**Case No.**	……
**Date (of sample receipt)**	/ /
**Name and Surname**	....
**Phone Number**	……………….
**1. Sociodemographic and Medical Characteristics**	
Age	(0-10 years: 1; 11-18 years: 2; 19-30 years: 3; 31-59 years: 4; 60+ years: 5)
Gender	(F: 1; M: 2)
Professional Status	(Student: 1; Unemployed: 2; Farmer: 3; Other: 4)
Nationality (Tunisian)	(No: 0; Yes: 1)
Governorate	……………………………………
Delegation	…………………………….
Nature of the Area	(Rural: 1; Urban: 2)
Medical History	....
**2. Exposure Characteristics**	
Contact with Animal	(No: 0; Yes: 1; Not mentioned: 2)
Date of Exposure Known	(No: 0; Yes: 1) If yes: / /
Animal Species	(Dog: 1; Other: ………………….)
If Dog - Ownerless	(No: 0; Yes: 1)
Vaccination Status of Animal	(Vaccinated: 1; Not vaccinated: 2; Unknown: 3)
Nature of Contact	(Bite: 1; Multiple bites: 2; Scratches: 3; Licking: 4; No exposure: 5)
Location of Bite (Single)	(No: 0; Yes: 1) If yes: (Head/Neck: 1; Upper limbs: 2; Lower limbs: 3; Other: 4)
Animal Diagnosed in Lab	(No: 0; Yes: 1) If yes, result date:

Field	Details
**3. Post-Exposure Prophylaxis**	
Medical Care Sought	(No: 0; Yes: 1; Not reported: 2)
Was Water and Soap Used for Washing	(No: 0; Yes: 1)
Visited Anti-Rabies Center	(No: 0; Yes: 1) If no, specify:
Time Between Exposure and Seeking Medical Care (days)	……………….
Treatment Received	(Vaccine: 1; Vaccine + Serum: 2)
Detailed Description of Protocol Received	………………………………………………………………………………
PEP Completed	(No: 0; Yes: 1)
If No, How Many Doses	……………………………………
PEP Done Correctly	(No: 0; Yes: 1)
If No, Why	………………………………………………………………………………
**4. Clinical Evolution Toward Death**	
Incubation Period (days)	……….
Symptoms Developed	Hydrophobia (No: 0; Yes: 1); Aerophobia (No: 0; Yes: 1); Hypersalivation (No: 0; Yes: 1);<br>Behavioral Changes (No: 0; Yes: 1); Paralysis (No: 0; Yes: 1); Fever (No: 0; Yes: 1)<br>Headache (No: 0; Yes: 1); Dysphagia (No: 0; Yes: 1); Arthralgia/Myalgia (No: 0; Yes: 1)
Other Symptoms	………………………………………………………………………………
Medical Structure Where Clinical Diagnosis Was Made	(Primary care: 1; Regional hospital: 2; Tertiary hospital: 3; Forensic service: 4)
Biological Diagnosis Performed in	(Ante-mortem: 1; Post-mortem: 2)
Time Between Clinical Diagnosis and Sample Collection	…………

Field	Details
Nature of Samples	(Brain tissue: 1; Corneal impression: 2; Saliva: 3; CSF: 4; Serum: 5; Other: 6)
Technique Used	(IFD: 1; IVCC: 2; PCR: 3; FAVN: 4; RFFIT: 5)
Time Between Sample Collection and Biological Confirmation (days)	……………….
Time Between Symptom Onset and Death (days)	……....
**Other Remarks**	....
